# Impact of using XP-endo finisher and nanobubble water during EDTA dentin conditioning on TGF-β1 release in regenerative endodontic procedures

**DOI:** 10.1186/s12903-024-04355-x

**Published:** 2024-05-22

**Authors:** Mai Sayed Hanafy, Ahmed Khaled Abdella Ahmed, Rana Gehad Salem

**Affiliations:** 1 Endodontic Department, Faculty of Dentistry, Suez University, Suez, Egypt; 2 Civil Engineering Department, Faculty of Engineering, Sohag University, Sohag, Egypt; 3 Pediatric Dentistry Department, Faculty of Oral and Dental Medicine, Badr University in Cairo, Cairo, Egypt

**Keywords:** Regenerative endodontic procedures, Nanobubble water, XP-endo Finisher, Growth factors, TGF-β1, Nanotechnology

## Abstract

**Introduction:**

Transforming Growth Factor-Beta 1 (TGF-β1) plays a crucial role in the success of Regenerative Endodontic Procedures (REPs) as they directly impact the proliferation and differentiation of stem cells. TGF-β1 is released by conditioning of the dentin matrix using 17% EDTA. EDTA was found to have deleterious effects on dentin especially in immature teeth with fragile dentin walls. Decreasing the irrigation time was reported to decrease these effects. Accordingly, enhancement and activation of the EDTA solution to maintain its efficiency in TGF-β1 release from dentin and thus compensating the reduction in irrigation time was employed. EDTA solution was enhanced by adding Nanobubble (NB) water which contains oxygen filled cavities less than 200 nm in diameter. Additionally, EDTA was activated with XP-endo Finisher rotary file. The aim of this study was to assess the impact of NB enhancement and/or XP-endo Finisher activation of the EDTA solution on the TGF-β1 release from dentin.

**Methods:**

Fifty standardized root segments with open apex were allocated to two main groups according to whether EDTA was enhanced with NB water or not, and within each group whether XP-endo Finisher activation was used or not in addition to a Negative Control group. The concentration of the released TGF-β1 in the root canal was measured using enzyme-linked immunosorbent assay (ELISA). The statistical analysis was done using the Shapiro- Wilk, Kolmogorov Smirnov, ANOVA and Post-hoc Tukey tests.

**Results:**

All groups released a considerable amount of TGF-β1 with the highest values in the EDTA/NB/XP group, followed by EDTA/NB, EDTA/DW/XP, EDTA/DW and Negative Control groups respectively.

**Conclusions:**

The results of this study suggest that NBs can promote the success of REPs since it revealed a significant increase in the TGF-β1 release following its use in the enhancement of the EDTA solution. A comparable effect was obtained by XP-endo finisher activation of the EDTA solution. The combined use of NBs and XP-endo Finisher can be a promising addition in REPs. Accordingly, Enhancement and activation of the EDTA solution may compensate decreasing the EDTA irrigation time attempted to avoid the deleterious effect of EDTA on dentin.

## Introduction

Treatment of non-vital open apex permanent teeth presents a tremendous challenge and requires collaborative efforts between the endodontic and pediatric specialties. The traditional conventional root canal treatment and apexification protocols routinely used do not result in the continuation of root maturation. Several trials were conducted to achieve the optimum goals of increasing the root length and root wall thickness as well as dentin-pulp complex regeneration [1–3]. 

In 2001, young permanent teeth were first reported to have the potential of revascularization following complete disinfection of the canals leading to complete apical closure as well as an increase in the thickness of the canal walls [4]. In 2007, the American Association of Endodontics (AAE) adopted the term “Regenerative Endodontic Procedures” (REPs) [5] to describe the concept of introducing endogenous stem cells inside the root canal after proper disinfection then placement of a fluid-tight coronal seal to prevent microleakage. The introduced stem cells result in the differentiation of odontoblast-like cells by the action of the growth factors embedded in the matrix of the root dentin and hence continuation of root maturation occurs [6–8]. 

REPs vary considerably in literature, but a key factor for their success is profound root canal disinfection through different irrigation protocols to eliminate the bacterial load thus allowing healing of the periradicular tissues [9, 10]. The irrigation protocol recommended by the American Academy of Endodontics (AAE) for regenerative procedures is irrigating the canals by 20 ml of sodium hypochlorite (NaOCl) 1.5% for 5 min followed by dentin conditioning with 20 ml of ethylenediaminetetraacetic acid (EDTA) 17% for 5 min [3]. 

Another crucial element of REPs success is the action of the growth factors. Growth factors directly impact the proliferation of stem cells and their differentiation which is an essential process for the dentin-pulp complex regeneration. Transforming Growth Factor-Beta 1 (TGF-β1), in particular, has a chemotactic action that facilitates stem cell migration into the root canals [11]. Additionally, it mediates signaling the proliferation, differentiation and mineralization of odontoblasts [2, 10, 12]. Growth factors are stored in dentin, and their release is triggered by conditioning and demineralizing the dentin matrix with chelating agents like EDTA, citric acid, phosphoric acid, and chitosan [6, 11, 13–16]. However, EDTA 17% has showed superior results regarding the growth factors’ release [17]. 

Although EDTA provides efficient smear layer elimination and organic material dissolution in addition to its action in growth factors release [18], it was found to have deleterious effects on dentin when used for extended periods of time or with high concentrations. These detrimental effects, which include dentin microhardness reduction, dentin matrix destruction, dentinal erosions, and subsequently decreased tooth fracture resistance [18–21], can be very critical particularly in immature teeth with thin dentinal walls. In order to minimize the weakening effect that EDTA has on dentin, reducing the irrigation time from 5 min to 3 min was proposed [20]. To compensate for the lower irrigation time and avoid compromising the amount of the growth factor released from the root dentin, EDTA enhancement and activation was sought out in the current study.

Recently, nanotechnology has been introduced to numerous dental, medical, and pharmaceutical disciplines. In the dental field, nanotechnology and nanomaterials were implemented in preventive dentistry, restorative dentistry, local anaesthesia, impression materials and implants [22]. Nanobubbles (NBs) are cavities filled with gas such as air, ozone, oxygen, nitrogen, carbon dioxide and hydrogen in aqueous solutions with diameter less than 200 nm that are produced by nanobubble generators [23, 24]. NBs have several unique properties such as their stability against collapse or burst, high internal pressure, charged surface and their ability to persist for weeks [25–28]. NBs have numerous applications in many domains such as chemical, agricultural, industrial, and environmental fields. In the medical field, NBs have been employed in intracellular drug delivery, increasing drug susceptibility in cancer cells, mineral processing, and surfactant-free cleaning [24]. In the dental field, NB water was used as an antibacterial agent to treat periodontal disease [29–31]. 

The use of NBs is promising in REPs through their addition to root canal irrigants thus enhancing their effectiveness by increasing their penetration and disinfection of the dentinal tubules, enhancing the smear layer removal, and promoting growth factor release. Preparation of the NaOCl irrigant using NB water was reported to improve its disinfection ability [32]. Also preparation of the EDTA irrigant using the NB water resulted in improving the growth factor release from dentin [7]. 

NBs have several proposed mechanisms of action such as adhering to solid materials and coalescing to form microbubbles that act as a wedge [32]. Also, the pressurised air inside the NBs may cause pressure waves exerting a kinetic force that removes fine particles from the root canal surface and dislodges the biofilm [32, 33]. Furthermore, NBs enhance the delivery of the irrigants by decreasing their surface tension and increasing their wettability [32]. 

EDTA activation was reported to promote growth factor release from dentin [13, 34]. Various strategies for irrigation activation is reported in literature including ultrasonic activation, laser activation, self-adjusting file system, Endo-Activator, photo-induced photoacoustic streaming, and XP-endo Finisher [7, 9, 13, 35, 36]. XP-endo Finisher is a rotary file that is claimed to provide optimal cleaning and disinfection of root canal by reaching canal spaces that were not accessible by standard instrumentation [37]. This is coupled with its ability to disturb the bacterial biofilm while preserving dentin [9]. 

The rationale of this study was to reduce the irrigation time with EDTA to minimize the detrimental effects that EDTA may have on dentin, particularly in immature teeth with fragile dentinal walls. In order to compensate for the reduction in irrigation time and avoid compromising TGF-β1 release from dentin, enhancing and activating the EDTA solution was employed. Hence, the aim of this study was to assess the impact of NB water enhancement of the EDTA solution and its activation with the XP-endo Finisher during dentin conditioning on the concentration of TGF-β1 released from dentin.

## Methods

The Research Ethics Committee, Suez University, Egypt approved this research with Approval Number #11,023. The total sample size of 50 teeth (10 teeth in each group) with an effect size of 0.35 was sufficient to acquire a power of 0.85 and a significance level of 0.05. The sample size was calculated using G*Power open-source software (Version 3.1, Universität Düsseldorf, Düsseldorf, Germany).

**Fig. 1:**
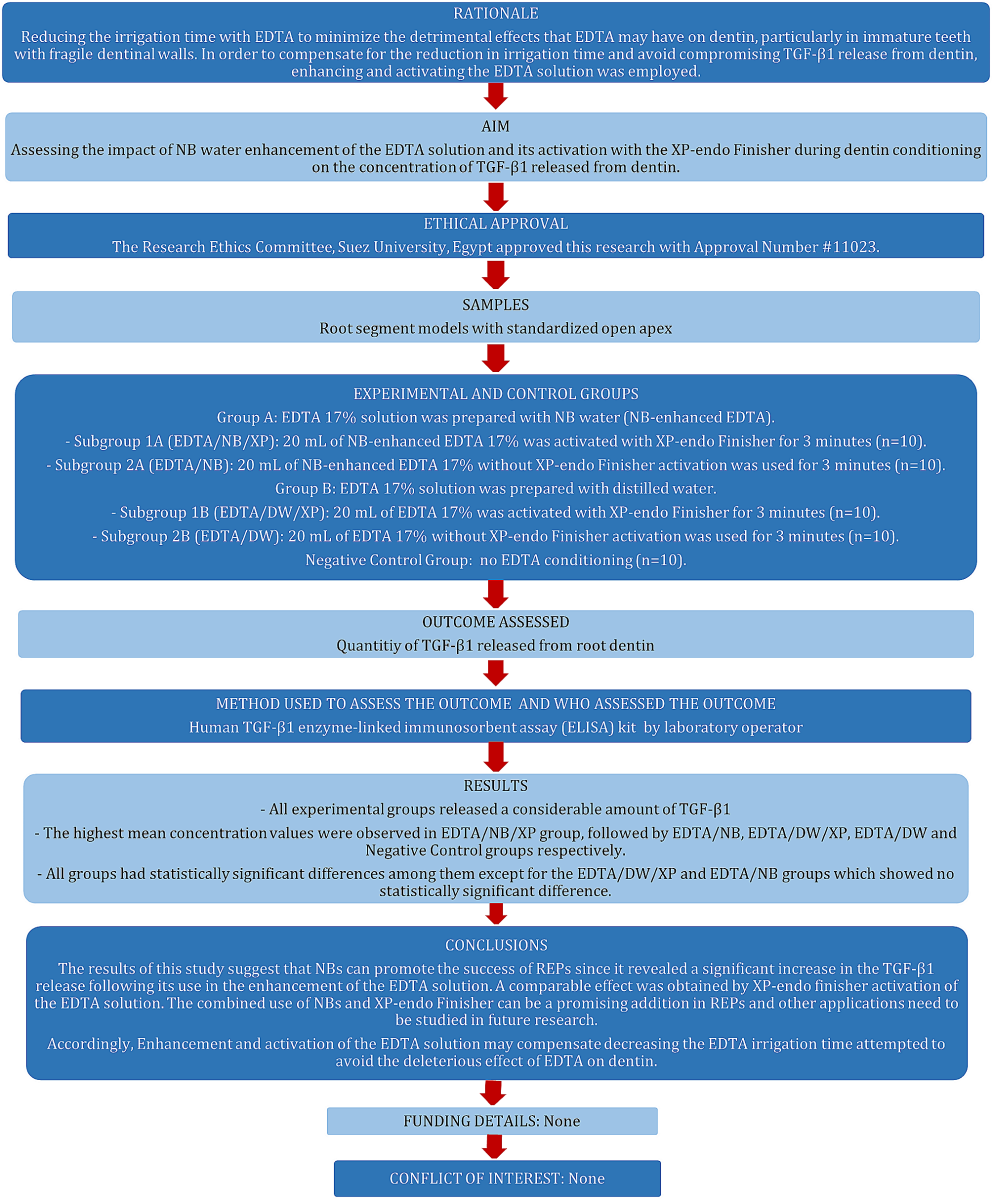
The study design according to PRILE guidelines

This study was written according to the Preferred Reporting Items for Laboratory studies in Endodontology (PRILE) guidelines 2021 [38], and the study design is described in the PRILE flowchart. (Fig. 1)

### Root segment model preparation

A total of fifty single-rooted mature permanent teeth were obtained from patients (17 to 40 years old) after having their informed consent to use their extracted teeth in this research work. Teeth included in the study were freshly extracted without any caries, resorption, cracks, fractures, developmental anomalies, or previous endodontic treatment and immediately preserved in saline solution (Sodium Chloride 0.9%, Al Mottahedoon Pharma, Egypt). Teeth cleaning was done using an ultrasonic scaler (Varios 550, NSK, Nakanishi, Japan); scrapping the outer tooth surface using a periodontal curette to remove any periodontal tissues was also done. Then, teeth were rinsed with sterile saline and stored until use.

Root segment models were prepared by decoronating teeth using sterile burs under water coolant and the remaining roots were standardized to 12 ± 1 mm height segment [39]. Stainless steel K-files size up to 100 (Mani Inc., Tokyo, Japan) were used in root canals to create a standardized open apex model with an apical diameter of 1 mm. After saline irrigation, nail polish was applied to every surface of the root except the inner surface, which was left uncovered in the experimental groups. In the Negative Control group, however, every root surface was coated.

Afterwards, irrigation of the root segments with 20 mL 1.5% NaOCl (Clorox, Egyptian Company for household products – under license of Clorox Co., USA – A.R.E, Egypt) was done for 5 min followed by 5 mL saline using side-vented needles (0.3 × 25, ENDO-TOP^®^, Cerkamed, Stalowa Wola, Poland).

### Randomization and blinding

Each irrigation protocol used in this study was coded, divided into groups, and then sealed inside an opaque envelope. A random sequence was generated using computer software (http://www.random.org/). The operator was aware of the type of protocol used at the time of irrigation since the used protocol must be exposed and cannot be masked while performing the procedure. The laboratory operator, data collector and the data analyst who conducted the statistical analyses were blinded to the irrigation protocol used to treat each group and subgroup.

### Irrigation protocols

Specimens were randomly allocated to two main groups according to whether EDTA was enhanced with NB water or not, and within each group whether XP-endo Finisher activation was used or not in addition to a Negative Control group as follows:

- Group A: EDTA 17% solution was prepared with NB water (NB-enhanced EDTA).
  - Subgroup 1 A (EDTA/NB/XP): 20 mL of NB-enhanced EDTA 17% was activated with XP-endo Finisher for 3 min (*n* = 10).
  - Subgroup 2 A (EDTA/NB): 20 mL of NB-enhanced EDTA 17% without XP-endo Finisher activation was used for 3 min (*n* = 10).
- Group B: EDTA 17% solution was prepared with distilled water.
  - Subgroup 1B (EDTA/DW/XP): 20 mL of EDTA 17% was activated with XP-endo Finisher for 3 min (*n* = 10).
  - Subgroup 2B (EDTA/DW): 20 mL of EDTA 17% without XP-endo Finisher activation was used for 3 min (*n* = 10).
- Negative Control Group: no EDTA conditioning (*n* = 10).

The irrigation procedure was performed under strict aseptic conditions in a laminar flow hood (Thermo Scientific™ 1300 Series Class II, Type A2- Thermo Fisher Scientific Inc. Ohio, USA). The room temperature irrigation solutions were dispensed using side-vented needles (ENDO-TOP^®^, Cerkamed, Stalowa Wola, Poland). During irrigation, the needle tip was placed inside the canal at about 1 mm short of the working length and moved in an upward and downward movement of about 3 mm coronal to this length and down back.

### NB water generation

**Fig. 2:**
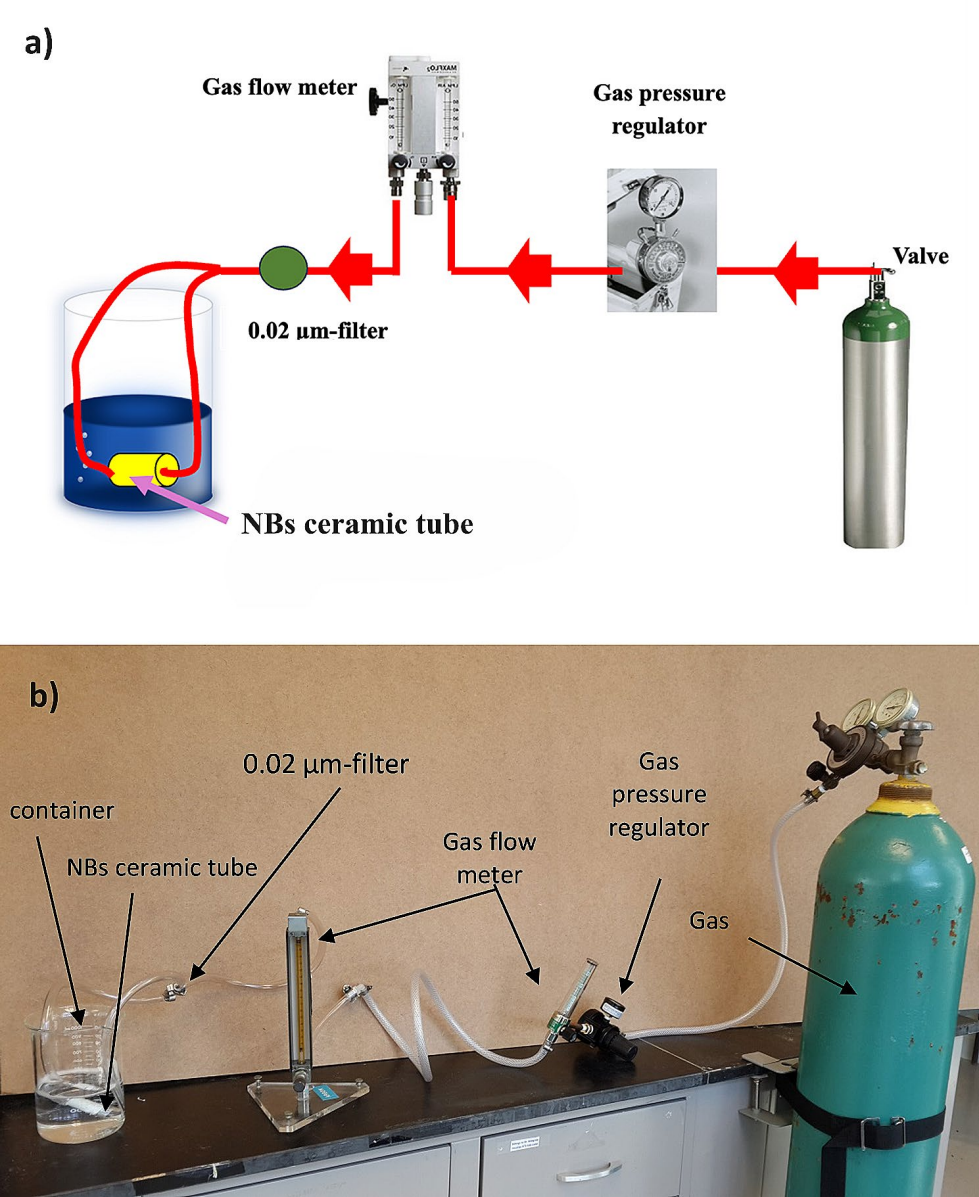
The NB generator system is composed of gas cylinder, gas pressure regulator, gas flow meter, 0.02 μm filter, ceramic tube filter and a container. **(a)** Schematic diagram of the NB generator system, **(b)** The bench top set up

NB water was prepared in a NB generator system (Fig. 2) Oxygen NBs are created by injecting compressed 99.99% pure oxygen gas from a gas cylinder through a gas flow meter and adjusted to 60 psi pressure using a gas pressure regulator. The pressurized gas then passes through a 0.02 μm filter (Whatman Anotop 25 Plus syringe filter, Sigma Alorich, USA) to eliminate all potential impurities in the gas flow. The gas then passes through a ceramic tube filter (Model WFA0.1, Refraction USA) with length of 51 mm, outer diameter 13 mm, inner diameter 8 mm and 100 nm nominal pore size into a 500 ml container containing Direct-Q® UV MilliporeⓇ purified water (Water purification system, Merck Millipore Inc., Darmstadt, Germany) [25, 26]. 

The hydrodynamic diameter of the resultant NBs was stabilized around 160 nm with a stable zeta potential (ZP) of -38 mV and around 40 mg/l of dissolved oxygen in the NB water.

### EDTA preparation

EDTA 17% was freshly prepared by dissolving 17 gm Ehylenediaminetetraacetic acid disodium salt dihydrate powder (Piochem, Laboratory Chemicals, Egypt) in 100 mL either distilled water (Pharmapack, Pharmaceutical Industries, Egypt) or NB water. The EDTA solution was adjusted to pH 7.4 by adding 1 M of sodium hydroxide.

### XP-endo finisher activation

The EDTA solution was activated by the XP-endo Finisher (0.0/25 NiTi Max Wire - FKG Dentaire, La Chaux-de-Fonds, Switzerland) by placing the file tip in the canal within 1 mm short of the working length and 7–8 mm vertical movement was done. The file was operated at 800 rpm speed and 1 N/cm maximum torque according to the manufacturer’s instructions. The working time was 1 min for 3 cycles (a total of 3 min activation time).

The root canals had a final saline flush of 5 mL after completing all irrigation and activation procedures, and finally dried with size #80 paper points (Meta Dental Co. Ltd., Chongju city, Korea).

### Sample collection and TGF-β1 quantification

The root segments were put into a microcentrifuge tube with 1 mL α-Minimum Essential Medium (α-MEM) (Gibco, Thermoscientific, USA) in addition to 1% penicillin/streptomycin (Gibco, Thermosientific, USA). Samples were then put in the incubator at 37^o^C for 24 h. Afterwards, the tubes were mixed well by vortex for 30 s then the collected medium was placed in 96-well plate (100µL/well sample). Quantifying the TGF-β1 released was done using Human TGF-β1 enzyme-linked immunosorbent assay (ELISA) kit (Cat no: EH0287, FineTest, Wuhan, China) following the manufacturer’s instructions. A spectrophotometer with a microplate ELISA reader (TC-98 ELISA reader, TECO Diagnostics, USA) was utilized to measure the optical density absorbance (450 nm) and the resulting values were compared with the ELISA kit standards.

### Determination of TGF-β1 concentration in the root canal

**Fig. 3:**
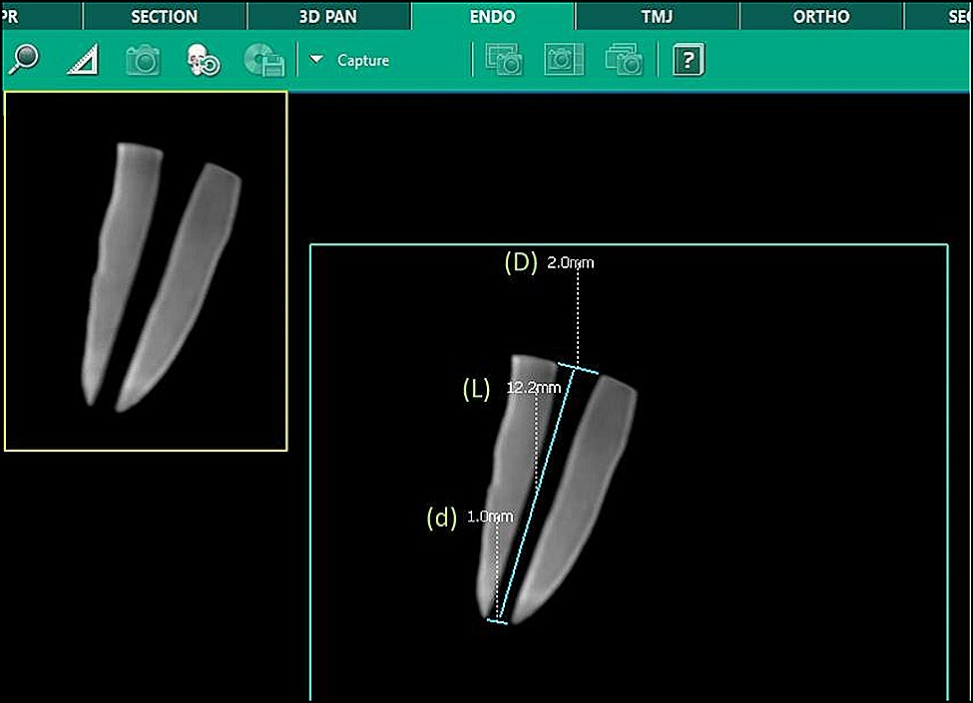
Post-preparation CBCT showing Length (L), Apical Diameter (d), and Coronal Diameter (D) of the root canal used for canal space volume (V_(Canal)_) determination

Each root segment had the prepared root canal volume measured using cone-beam computed tomography (CBCT) (Vatech Green CT, Vatech Co. Ltd., Hwaseong-si, Korea) by utilizing a 5 × 5 cm height field size, 4.9 s exposure time, 99 kV, 16 mA and 0.125 mm slice thickness. Measuring the root canal’s length as well as both the coronal and the apical diameters was done using the Ez3D-i dental software (3D Software, Vatech Co. Ltd., Hwaseong-si, Korea) in a sagittal view (Fig. 3).

The canal space volume (V_(Canal)_) was determined by the equation; “V_(Canal)_ = π*L{(D/2)^2^ + (D/2)(d/2) + (d/2)^2^/3}” and the final TGF-β1 concentration volume (C_(Canal)_) in pg/mL was determined by the equation; “C_(Canal)_ = C_(ELISA)_ x V_(collecting medium)_/V_(canal)_” [39]. 

## Results

**Fig. 4:**
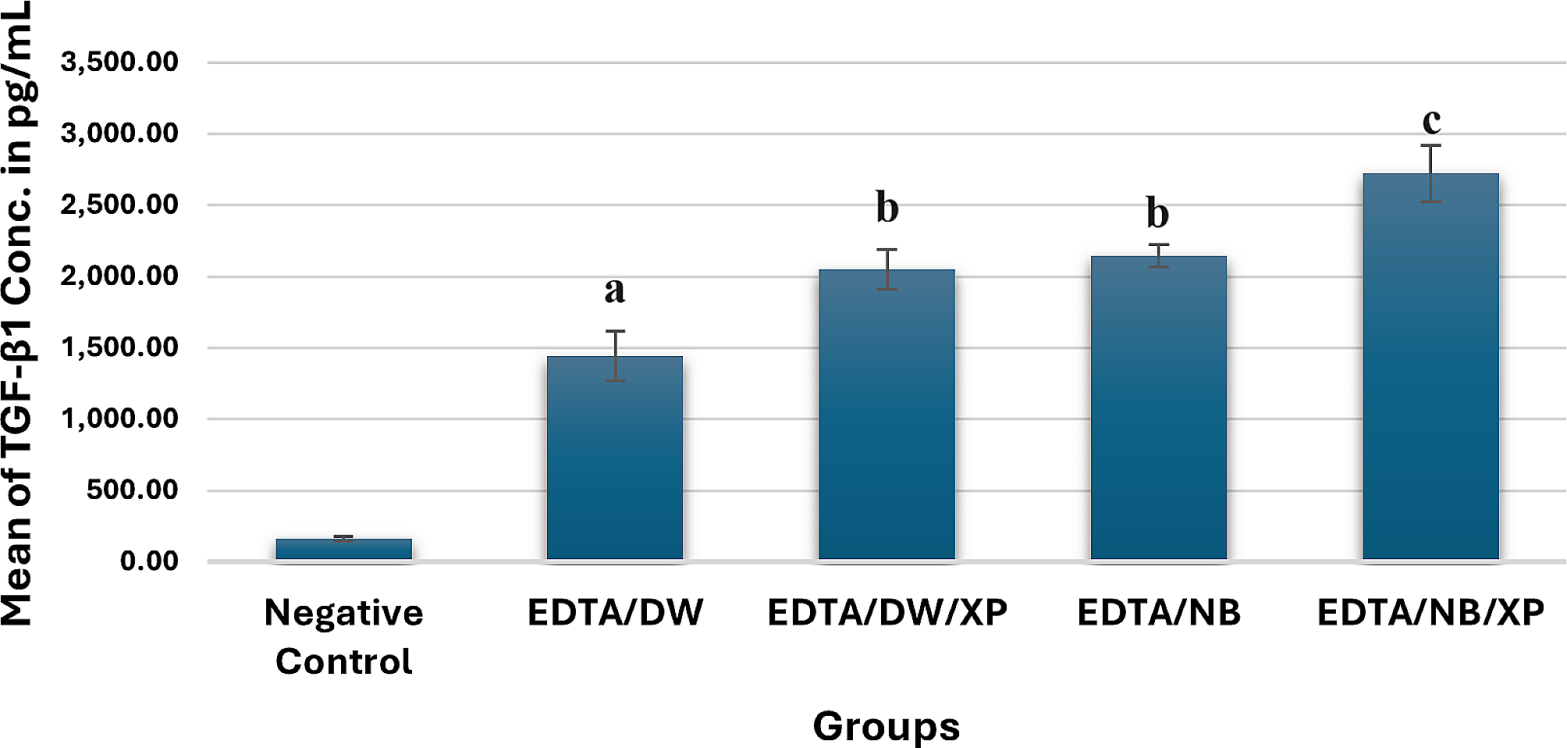
Bar chart showing the mean TGF-β1 concentration (pg/mL) results in different groups. Different letters indicate statistically significant differences among groups and when compared with the control group (*p-value* < 0.05)

TGF-β1 volume concentration data were collected, and the statistical analysis was done utilizing the SPSS Statistics software (Version 27.0 - IBM Corp, Armonk, NY, USA). To indicate any statistical difference among the tested groups, a *p*-value < 0.05 significance level was established. The Shapiro- Wilk and Kolmogorov Smirnov tests were used to test the continuous variables for normality. One-way analysis of variance (ANOVA) test and Post-hoc Tukey test were used to compare the mean values of TGF-β1 concentration and to perform comparisons between the groups respectively. Descriptive statistics of TGF-β1 concentration (release) values for each group are expressed in (Table 1) and (Fig. 4).

**Table 1:** Descriptive statistics for the TGF-β1 concentration values (pg/mL) in different groups

**Groups**	**Mean ± SD**	**95% CI for Mean**		**Min**	**Max**	***p-value***
		**Lower** **Bound**	**Upper** **Bound**			
**Negative Control**	161.15 **±** 24.60	143.55	178.75	130.12	194.75	< 0.001
**EDTA/DW**	1443.38 **±** 244.84 ^a^	1268.23	1618.52	1175.60	1801.74	< 0.001
**EDTA/DW/XP**	2050.13 **±** 194.51 ^b^	1910.98	2189.27	1786.72	2317.99	0.817
**EDTA/NB**	2143.05 **±** 107.77 ^b^	2065.96	2220.15	1926.65	2264.49	0.817
**EDTA/NB/XP**	2722.10 **±** 275.75 ^c^	2524.84	2919.36	2273.12	3069.89	< 0.001

Different letters indicate statistically significant differences among irrigation protocols and when compared to control (*p-value* < 0.05).

The mean volume of the prepared root canal space was found to be 22.93 ± 1.95 mm^3^.

Although all experimental groups released a considerable amount of growth factor (TGF-β1) (pg/mL); however, the highest mean concentration values were observed in EDTA/NB/XP group (2722.10 ± 275.75), followed by EDTA/NB (2143.05 ± 107.77), EDTA/DW/XP (2050.13 ± 194.51), EDTA/DW (1443.38 ± 244.84) and Negative Control (161.15 ± 24.60) groups respectively.

All groups had statistically significant differences among them (*p*-value < 0.001) except for the EDTA/DW/XP and EDTA/NB groups which showed no statistically significant difference (*p*-value = 0.817).

## Discussion

REPs address the continuation of root maturation of immature permanent teeth that lost their vitality. The main aspects contributing to the success of REPs are profound canal disinfection, introduction of stem cells into the canal space with the presence of a scaffold and growth factors released from the dentin matrix.

TGF-β1 is a growth factor released from dentin after conditioning of the dentin matrix using 17% EDTA. EDTA was found to have deleterious effects on dentin, so decreasing the irrigation time was suggested in an attempt to decrease this effect. Accordingly, enhancement and activation of the EDTA to maintain its efficiency was employed.

This in-vitro study assessed enhancing the EDTA solution with NB water and its activation with the XP-endo Finisher and compared their effect on TGF-β1 release from dentin while reducing the EDTA irrigation time.

In this investigation, the root segment model was employed by decoronating the teeth to a 12 ± 1 standard length in accordance with several studies [13, 15, 39]. Using the root segment model is more closely aligned with the clinical scenario, since the release of growth factors occurs from the inner dentin surface exclusively [17]. Other studies used the dentin disc model which may produce inaccurate results because growth factors are released from both the inner and outer dentin surfaces [10, 40–42]. Root segments were hand instrumented up to size #100 K-file in order to create a standardised, truncated, cone-shaped canal with a 1 mm open apical diameter which was reported to be the critical apical diameter in REPs [43, 44]. 

The irrigation protocol adopted in this study was NaOCl 1.5% followed by EDTA 17% following the recommendations of the AAE for regenerative procedures [3]. The irrigation time was decreased to 3 min [20] instead of the 5 min recommended by the AAE [3] since some studies discussed the adverse effects of prolonged exposure and high concentration of the EDTA solution on the dentin microhardness, integrity, and structural properties as well as the reduction of the tooth resistance to fracture which can be critical especially in immature teeth [18–21]. 

In an attempt to compensate the lower irrigation time and to avoid compromising the amount of the growth factor released from the root dentin, EDTA enhancement and activation using NB water and XP-endo Finisher was employed.

TGF-β1 was quantified using ELISA following the canal irrigation procedure. This approach aligns with prior research, as measuring growth factors post-irrigation mirrors the clinical scenario where bleeding is induced at the apex after irrigation and drying [6, 14, 45]. This differs from the study by Galler et al., where growth factor release was measured during irrigation [10]. 

Our results showed a statistically significant higher TGF-β1 release in both EDTA/DW/XP and EDTA/NB groups compared to EDTA/DW group. The superior results in the EDTA/NB group may be attributed to the properties of the NBs as well its mechanism of action. The size of the NBs in our study was stabilized around 160 nm and with a -38 mV stable ZP and around 40 mg/l of dissolved oxygen in the NB water. The size of our NBs conforms with the size of the NBs reported in a study by Shawli in 2020 that demonstrated improved removal of smear layer when using 17% EDTA enhanced by NB water compared to EDTA alone [32]. 

It was proposed that NBs reduce the surface tension of the irrigants and increase their internal pressure creating pressure waves that eliminate small particles from the surface of solid materials [7, 32, 33]. Thus, NBs were shown in several studies to promote the disinfection capacity of irrigants, enhance smear layer removal, and improve the irrigants penetration into the dentinal tubules [7, 32, 33, 46]. The effect of EDTA prepared with NB water on TGF-β1 release was reported in one study and found to exert a little effect but that study reported a limitation of not specifying the NBs size which may affect its properties [7]. 

On the other hand, in the EDTA/DW/XP group, the positive effect of the EDTA activation using the XP-endo Finisher can be attributed to enhanced smear layer removal and thereby reaching inaccessible areas while preserving dentin. Moreover, the XP-endo Finisher has improved irrigation dynamics as the file exhibits increased flexibility and enhanced expansion capacity which leads to the improvement of the penetration of the irrigant into the dentinal tubules [45]. The increase in the TGF-β1 release in the EDTA/DW/XP group is in accordance with studies that showed that irrigation activation increases the growth factor release from dentin [7, 13, 34, 47]. XP-endo Finisher showed higher results when compared with passive ultrasonic irrigation during activation of the irrigants on smear layer removal and dentinal tubules opening as well as increased bacterial removal [48, 49]. 

The EDTA/NB/XP group showed the highest levels of released TGF-β1 which may be attributed to a boost in the NBs dynamics by the XP-endo Finisher agitation. A synergistic effect was reported in a study that examined the impact of ultrasonic agitation and microbubble emulsion on endodontic biofilm and attributed the beneficial outcome to the coalescing of the NBs together forming larger bubbles as well as creating a turbulent irrigant flow along the walls of the canal [50]. However, more studies may be needed to clarify this synergistic effect.

As for the limitations of the current study, the TGF-β1 release was assessed at 3 min EDTA irrigation time but it wasn’t compared with the 5 min irrigation time recommended by the AAE which can be considered in future studies. The results of the current study could be a starting point for future research on other applications of NBs in REPs such as canal disinfection, smear layer removal and stem cells viability. Furthermore, the in vivo clinical feasibility of NBs can be examined and compared to the XP-endo Finisher.

## Conclusions

The results of this study suggest that NBs can promote the success of REPs since it revealed a significant increase in the TGF-β1 release following its use in the enhancement of the EDTA solution. A comparable effect was obtained by XP-endo finisher activation of the EDTA solution. The combined use of NBs and XP-endo Finisher can be a promising addition in REPs and other applications need to be studied in future research. Accordingly, Enhancement and activation of the EDTA solution may compensate decreasing the EDTA irrigation time attempted to avoid the deleterious effect of EDTA on dentin.

## Data Availability

The data that support the findings of this study are available from the corresponding author upon reasonable request at (rana.gehad@buc.edu.eg).

## Abbreviations

AAE: American Association of Endodontics
CBCT: Cone-beam computed tomography
EDTA: Ethylenediaminetetraacetic acid
ELISA: Enzyme-linked immunosorbent assay
NaOCl: Sodium hypochlorite
NB: Nanobubble
REPs: Regenerative Endodontic procedures
TGF-β1: Transforming Growth Factor-Beta 1
ZP: Zeta potential
α-MEM: α-Minimum Essential Medium
